# The Book World of Medicine and Science

**Published:** 1903-08-29

**Authors:** 


### The Book World of Medicine and Science.

Tumours, Innocent and Malignant: their Clinical Characters and Appropriate Treatment. By J. Bland-Sutton. (Third edition. With 312 engravings. Pp. 556. Cassell and Company. 1903. 21s.)

Mr. Bland-Sutton's copiously illustrated work on tumours, which was published ten years ago and has been twice reprinted, has now been revised and considerably added to, both in the text and the illustrations. It keeps its distinctive character as a work combining a fair amount of pathology with more of the picturesque and practical aspects of tumours. The histological discussion is still subordinate in this edition, but a number of microscopic figures have been introduced, including several excellent ones bearing the initials "L. W. B.". Also the concise remarks upon minute structure may be depended upon as accurate, although they cannot be said to be profound, or to throw any new light upon the question of the beginnings of cancers, sarcomas, endotheliomas, etc., in their respective seats. The chapter on Myelomata, under which name Paget's myeloid tumours of bone are described, is clear and conclusive as well as beautifully illustrated. The same praise should be given to the other sections upon new growths of the bones, and to those upon fibroids, etc., of the body of the uterus and the appendages, upon dermoids, neuromata, and others. The wide range of his matter will appear from the fact that he has no fewer than sixty chapters, under many of which one finds curious and interesting illustrations from development and from the lower animals. The author's object being to survey the whole phenomena of tumours so as to bring out their remarkable variety and richness of forms, it is not to be expected that he should occupy space with the cancers of the cervix uteri, pylorus, breast, and rectum in the ratio of their importance. He has been, indeed, as liberal to the innocent as to the malignant, and he expresses the opinion in his new preface "that the causes of the so-called benign tumours are as mysterious as of those that are malign." It is a useful reminder in one way; but as Mr. Bland-Sutton himself has done much to make the innocent tumours intelligible from the embryological point of view, his phrase "as mysterious" should mean no more than that both kinds are alike intricate and recondite in respect of their origins at certain parts or from certain tissues. On the point of malignancy itself no tumours are more "deadly," as the author says, than the sarcomas of growing bones, both internal and periosteal; but their physiological type is not less clear than that of the myelomata, which are so nearly free from malignancy that he separates them from the other internal sarcomas of bone. One of his new sections is on Deciduoma (of which the discoverer was Professor Rudolf Maier, and not the two gynæcologists mentioned at p. 76). Its resemblance to placental syncytium is striking, and yet it has a boring and rodent propensity which makes it highly malignant. On the much-debated question of free and wide removal the author's practical course is decided, but his argument halting and weak. At p. 240 he writes: "It is a fact which every surgeon who has had much experience in operating for cancer must have noticed, that in some instances where he has conducted carefully-planned but extensive operations for cancer, the patient has had rapid recurrence, and the disease has manifested itself in a manner far worse than when left to run its natural course." An explanation follows, depending on the theory that cancer-cells are liberated and dispersed by the incisions and manipulations; and the way to obviate that result appears to be to make the extensive operation still more extensive. The unsatisfactory position in which the question is left is shown by the following sentence at p. 252: "Although surgery is in some of these cases fairly successful, yet, broadly reviewing the whole subject for the relief of cancer, we must admit that our present mode of treating it, namely, 'to cut out the diseased organ or part affected,' though extremely clumsy, is the only really effectual method as yet devised."

Manual of Medicine. By T. K. Munro. (London: Baillière, Tindall and Cox. 1903. Demy 8vo. Pp. 901. 39 Illustrations. 15s. net.)

This work, which now appears for the first time, and is published in the "University Series," supplies a want among English treatises of a convenient-sized manual sufficient for the needs of the modern student of medicine. The book is divided into twelve sections, and to the first, on Specific Infectious Diseases, there is given an excellent though brief introduction in the form of a consideration of the present views of the nature, pathology, and treatment of Fever. Each of the infectious diseases is dealt with seriatim, beginning with Typhus Fever; in all 41 varieties being described. The author follows Osler in classing Rheumatic Fever with the Infectious Diseases. Constitutional Diseases are described in Section 2. Other sections are devoted to the Cardio-vascular Diseases, Diseases of the Blood, Respiratory System, Digestive System, Kidneys, Nervous System, Muscles, and of the Skin; each section being a model of conciseness and method. Section 11 includes Intoxications and Sunstroke, and the last section a comprehensive and yet well-proportioned account of diseases due to animal parasites. The book is a thoroughly sound and up-to-date work on General Medicine. It is well written, produced in clear type, with good diagrams and a copious index. Alike to the student for final examination, and the general practitioner of medicine, we can confidently recommend Dr. Munro's book.

The Physiological Nursery Chart. Designed by Eric Pritchard, M.A., M.D.Oxon., M R.C.P.Lond. (Published by Henry Kimpton. Price 1s. 1d. post free.)

This chart is intended to be hung up in the nursery and has the form of the familiar commercial almanac issued by the enterprising tradesman towards the end of every year. It is printed on both sides, the centre of the front page consisting of two weight charts, the one from the first to the fifty-second week, the second on a smaller scale for the second and third years. There are also directions for feeding and weighing the child, with a list of symptoms, showing whether or not the food is assimilated. The dorso is headed "A Nursery Emergency Chart," and gives directions as to the course to be pursued in a case of sudden illness and in cases of poisoning, whilst the last column consists of 20 nursery aphorisms relating to the baby's hygiene. A dentition table and a series of tests of normal muscular development completes the information. The radical and all condemning defect in the chart is the fact that nowhere is it even hinted as to the proper food for an infant. The mother's milk is all-sufficient for a baby from the first and for many weeks, yet in the whole chart there is no mention of such a physiological diet. Circumstances may and often do render it necessary to feed a baby otherwise, but it is a very mischievous thing to issue a chart which is called physiological and is evidently designed for extensive circulation without any allusion at all to breast feeding. The facts contained in the chart are substantially accurate, and a great deal of information is compressed into the space. The tests of normal muscular development in an infant are interesting from many points of view. It is stated that at three to four months a baby should be able to hold up his head when his body is held erect, and at seven months he should be able to sit up unsupported for a few minutes.

